# Supplementary material for: Hybrid AI-assistive diagnostic model permits rapid TBS classification of cervical liquid-based thin-layer cell smears
Source: Nat Commun. 2021 Jun 10;12:3541. doi: 10.1038/s41467-021-23913-3 (PMC8192526; doi:10.1038/s41467-021-23913-3)
Supplement: Supplementary file 1 — Supplementary Information files [file 41467_2021_23913_MOESM1_ESM.pdf]

Supp. Table 01 The Bethesda System (2014) of cervical cytology

Abbreviation of TBS classification	TBS classification	Remarks
ASCUS	Atypical squamous cells of undetermined significance	Nil
LSIL	Low-grade squamous intraepithelial lesion	Nil
ASCH	Atypical squamous cells; cannot exclude high-grade intraepithelial lesion	Nil
HSIL	High-grade squamous intraepithelial lesion	Nil
SCC	Squamous cell carcinoma	Nil
AGC_NOS	Atypical glandular cells, not otherwise specified	Including atypical cervical gland cells, atypical endometrial cells and other atypical glandular cells
AGC_FN	Atypical glandular cells, favor neoplastic	Including atypical cervical gland cells and other atypical glandular cells
AIS	Adenocarcinoma in situ	Located in the cervix
ADC	Adenocarcinoma	Including cervical adenocarcinoma, endometrial adenocarcinoma, extrauterine adenocarcinoma and other unspecified adenocarcinoma
EMC	Endometrial cells	In women over 45 years old
TRI	Trichomonas vaginalis	Nil
CAN	Candida albicans	Nil
HSV	Herpes simplex virus	Nil
CC	Clue cell	Nil
ACTINO	Actinomycetes	Nil
NILM	Negative for intraepithelial lesion or malignancy	Normal

Supp. Table 02 Comparison of computational speed and accuracy between YOLOv3 model and other deep learning models in detecting squamous intraepithelial lesions

Methods	Backbone	mAP@0.5	Time (ms)
FASTER R-CNN W FPN	ResNet-101-FPN	85.6	172
SSD513	ResNet-101-SSD	80.9	125
RETINANET	ResNet-101-FPN	84.3	198
YOLOV3-608	Darknet-53	82.1	53

The mAP@0.5 represents the mean average precision when the Mean Intersection Over Union (mIOU) reaches 0.5 above during the training process. *ms*: milliseconds.

Supp. Table 03 Accuracy of 5-fold cross-validation of cell nucleus segmentation model

Fold	IOU @ ASC_L_S	IOU @ SC	IOU @ ASC_H_S	mIOU
1	0.8502	0.8231	0.8803	0.8512
2	0.8324	0.8453	0.9074	0.8617
3	0.8095	0.7886	0.8468	0.815
4	0.8389	0.7498	0.8611	0.8166
5	0.8921	0.7713	0.8379	0.8338
**Total**	**0.8446**	**0.7956**	**0.8667**	**0.8356**

IUO: intersection over union. mIOU: mean intersection over union

Supp. Table 04 Features of XGBoost model for squamous intraepithelial lesions

Methods	Feature ID	Annotation
Nucleus segmentation	S1	The mean value of ASC_L_S gray value / S24 (only include the ratio in top 50%)
Nucleus segmentation	S2	The number of ASC_L_S with gray value / S24 > 2
Nucleus segmentation	S3	The number of ASC_L_S with gray value / S24 > 2.5
Nucleus segmentation	S4	S3 / WC * 10^3
Nucleus segmentation	S5	S2 / WC * 10^3
Nucleus segmentation	S6	The number of ASC_L_S with gray value / S24 > 3
Nucleus segmentation	S7	S6 / WC * 10^3
Nucleus segmentation	S8	S2 + 1.5 * S3 + 2.5 * S6
Nucleus segmentation	S9	S8 / WC * 10^3
Nucleus segmentation	S10	S8 / 5 / N-ASC_L_S
Nucleus segmentation	S11	S3 / N-ASC_L_S
Nucleus segmentation	S12	S2 / N-ASC_L_S
Nucleus segmentation	S13	S6 / N-ASC_L_S
Nucleus segmentation	S14	The mean value of ASC_H_S gray value / S24 (only include the ratio in top 30%)
Nucleus segmentation	S15	The number of ASC_H_S with gray value / S24 > 2
Nucleus segmentation	S16	The number of ASC_H_S with gray value / S24 > 2.5
Nucleus segmentation	S17	S16 / WC * 10^3
Nucleus segmentation	S18	S15 / WC * 10^3
Nucleus segmentation	S19	S15 + 1.5 * S16
Nucleus segmentation	S20	S19 / WC * 10^3
Nucleus segmentation	S21	S19 / 5 / N-ASC_H_S
Nucleus segmentation	S22	S16 / N-ASC_H_S
Nucleus segmentation	S23	S15 / N-ASC_H_S
Nucleus segmentation	S24	the number of SC (0.5 ≤ SC gray value ≤ 1.5 the mean gray value of all SC)
Nucleus segmentation	S25	S24 / WC * 10^3
Nucleus segmentation	S26	the mean gray value of SC (0.5 ≤ SC gray value ≤ 1.5 the mean gray value of all SC)
Patch	P-L1	The median of Ture_LSIL probability (TLP) of ASC_L_S

Patch	P-L2	The median of False_LSIL probability (FLP) of ASC_L_S
Patch	P-L3	The median of TLP / FLP of ASC_L_S
Patch	P-L4	The median of TLP * (1 - FLP) of ASC_L_S
Patch	P-L5	The median of TLP - FLP of ASC_L_S
Patch	P-H1	The median of Ture_HSIL probability (THP) of ASC_H_B, ASC_H_M, ASCH_H_S and SCC_G
Patch	P-H2	The median of False_HSIL probability (FHP) of ASC_H_B, ASC_H_M, ASCH_H_S and SCC_G
Patch	P-H3	The median of THP / FHP of ASC_H_B, ASC_H_M, ASCH_H_S and SCC_G
Patch	P-H4	The median of THP * (1 - FHP)of ASC_H_B, ASC_H_M, ASCH_H_S and SCC_G
Patch	P-H5	The median of THP - FHP of ASC_H_B, ASC_H_M, ASCH_H_S and SCC_G
Patch	P-L6	The median of Ture_LSIL probability (TLP) of KC and ASC_L_F
Patch	P-L7	The median of False_LSIL probability (FLP) of KC and ASC_L_F
Patch	P-L8	The median of TLP / FLP of KC and ASC_L_F
Patch	P-L9	The median of TLP * (1 - FLP) of KC and ASC_L_F
Patch	P-L10	The median of TLP - FLP of KC and ASC_L_F
Combination	XY1	N-ASC_L_S + 2*(N-KC + N-ASC_L_F) + 2*(N-ASC_H_B + N-ASC_H_M) + N-ASC_H_S
Combination	XY2	XY1 / WC * 10^3
Combination	XP1	X-L4 * P-L3

Combination	XP2	X-L4 * P-L4
Combination	XY3	(p-N-ASC_L_S + p-N-ASC_L_S_0.2) / 2
Combination	XY6	2 * (N-ASC_H_B + N-ASC_H_M) + ASC_H_S + N-SCC_G + N-SCC_G_0.2 + N-SCC_R + N-SCC_R_0.2
Combination	XY7	XY6 / WC * 10^3
Combination	XP3	XY7 * P-H3
Combination	XP4	XY7 * P-H4
Combination	XY4	(p-N-ASC_H_S + p-N-ASC_H_S_0.2) / 2
Combination	XP5	X-L9 * P-L8
Combination	XP6	X-L9 * P-L9
Combination	XY5	(p-N-KC + p-N-KC_0.2) / 2
Combination	XY8	N-SCC_G + N-SCC_G_0.2
Combination	XY9	XY8 / WC * 10^3
Combination	XY10	N-SCC_R + N-SCC_R_0.2
Combination	XY11	XY10 / WC * 10^3
Combination	WC	The number of patches detected by yolov3 model in the WSI
Xception	X-L1	X-L2 / M-ASC_L_S_MC
Xception	X-L2	N-ASC_L_S / N-ASC_L_S_0.0264
Xception	X-L3	N-ASC_L_S
Xception	X-L4	N-ASC_L_S / WC * 10^3
Xception	X-L5	X-L4 * M-ASC_L_S_MC
Xception	X-L6	N-ASC_L_S + 2 * (N-KC + N-ASC_L_F)
Xception	X-L7	X-L6 / WC * 10^3
Xception	X-H1	1 - N-SC_XY / N-SC_X
Xception	X-H2	N-ASC_H_B
Xception	X-H3	N-ASC_H_B / WC * 10^3
Xception	X-H4	2 * (N-ASC_H_B + N-ASC_H_M) + N-ASC_H_S
Xception	X-H5	X-H4 / WC * 10^3
Xception	X-H6	N-ASC_H_M
Xception	X-H7	N-ASC_H_M / WC * 10^3
Xception	X-H8	X-H9 / M-ASC_H_S_SC
Xception	X-H9	N-ASC_H_S / N-ASC_H_S_0.0264
Xception	X-H10	N-ASC_H_S
Xception	X-H11	N-ASC_H_S / WC * 10^3
Xception	X-H12	X-H11 * M-ASC_H_S_SC

Xception	X-H13	p-N-ASC_H_S_0.2 + X-H1
Xception	X-L8	N-KC + N-ASC_L_F
Xception	X-L9	X-L8 / WC *10^3
Xception	X-L10	X-L11 / M-KC_MC
Xception	X-L11	N-KC / N-KC_0.0264
Xception	X-L12	N_KC
Xception	X-L13	N_KC / WC * 10^3
Xception	X-L14	N-ASC_L_F
Xception	X-L15	N-ASC_L_F / WC * 10^3
Xception	X-M1	M-ASC_L_S_MC
Xception	X-M2	M-KC_MC
Xception	X-R1	N-RC_0.9
Xception	X-R2	N-RC_0.9 / WC *10^3
Xception	X-R3	M-ASC_H_B and ASC_H_M_RC
Xception	X-RS1	X-R1^2 * N-SC
Xception	X-RS2	X-RS1 / WC * 10^6
Xception	X-SC1	N-SC
Xception	X-SC2	N-SC / WC * 10^3
Xception	X-SC3	N-SC / N-ASC_H_S
Xception	X-SC4	N-SC_XY / N-SC_X
Xception	X-SC5	M-ASC_H_S_SC
Xception	X-SCC1	N-SCC_G
Xception	X-SCC2	N-SCC_G / WC * 10^3
Xception	X-SCC3	N-SCC_R
Xception	X-SCC4	N-SCC_R / WC * 10^3
YOLOv3	Y-L1	p-N-ASC_L_S_0.2 / p-N-ASC_L_S
YOLOv3	Y-L2	N-ASC_L_S_0.0264
YOLOv3	Y-L3	N-ASC_L_S_0.0264 / WC * 10^3
YOLOv3	Y-L4	N-ASC_L_S_0.2
YOLOv3	Y-L5	N-ASC_L_S_0.2 / WC * 10^3
YOLOv3	Y-L6	p-N-ASC_L_S_0.2
YOLOv3	Y-H1	p-N-ASC_H_S_0.2 / p-N-ASC_H_S
YOLOv3	Y-H2	N-ASC_H_S_0.0264
YOLOv3	Y-H3	N-ASC_H_S_0.0264 / WC * 10^3
YOLOv3	Y-H4	p-N-ASC_H_S_0.2
YOLOv3	Y-L7	p-N-KC_0.2 / p-N-KC
YOLOv3	Y-L8	N-KC_0.0264
YOLOv3	Y-L9	N-KC_0.0264 / WC * 10^3
YOLOv3	Y-L10	p-N-KC_0.2

YOLOv3	Y-S1	N-SCC_G_0.0264
YOLOv3	Y-S2	N-SCC_G_0.0264 / WC * 10^3
YOLOv3	Y-S3	N-SCC_R_0.0264
YOLOv3	Y-S4	N-SCC_R_0.0264 / WC * 10^3

Appendix: Abbreviation

Name	Annotation
N-ASC_L_S	Number of ASC_L_S (with yolov3 probability ≥ 0.0264 and Xception probability ≥ 0.9)
N-ASC_H_S	Number of ASC_H_S (with yolov3 probability ≥ 0.0264 and Xception probability ≥ 0.9)
N-KC	Number of KC (with yolov3 probability ≥ 0.0264 and Xception probability ≥ 0.9)
N-ASC_L_F	Number of ASC_L_F (with yolov3 probability ≥ 0.0264 and Xception probability ≥ 0.9)
N-ASC_H_B	Number of ASC_H_B (with yolov3 probability ≥ 0.0264 and Xception probability ≥ 0.9)
N-ASC_H_M	Number of ASC_H_M (with yolov3 probability ≥ 0.0264 and Xception probability ≥ 0.9)
N-SC	Number of SC (with yolov3 probability ≥ 0.0264 and Xception probability ≥ 0.9)
N-SCC_G	Number of SCC_G (with yolov3 probability ≥ 0.0264 and Xception probability ≥ 0.9)
N-SCC_R	Number of SCC_R (with yolov3 probability ≥ 0.0264 and Xception probability ≥ 0.9)
N-ASC_L_S_0.0264	Number of ASC_L_S (with yolov3 probability ≥ 0.0264)
N-ASC_H_S_0.0264	Number of ASC_H_S (with yolov3 probability ≥ 0.0264)
N-KC_0.0264	Number of KC (with yolov3 probability ≥ 0.0264)
N-SCC_G_0.0264	Number of SCC_G (with yolov3 probability ≥ 0.0264)
N-SCC_R_0.0264	Number of SCC_R (with yolov3 probability ≥ 0.0264)
N-ASC_L_S_0.2	Number of SCC_R (with yolov3 probability ≥ 0.2)
N-SC_XY	Number of SC (consistent classification predicted by both yolov3 and Xception)
N-SC_X	Number of SC (only predicted as SC by Xception)
N-RC_0.9	Number of RC (with Xception probability ≥ 0.9)
M-ASC_L_S_MC	The median probability that ASC_L_S (with yolov3 probability ≥ 0.0264 and Xception probability ≥ 0.9) is MC in Xception model
M-ASC_H_S_SC	The median probability that ASC_H_S (with yolov3 probability ≥ 0.0264 and Xception probability ≥ 0.9) is SC in Xception model
M-KC_MC	The median probability that KC (with yolov3 probability ≥ 0.0264 and Xception probability ≥ 0.9) is MC in Xception model
M-ASC_H_B and	The median probability that ASC_H_B and ASC_H_M (with yolov3

ASC_H_M_RC	probability ≥ 0.0264 and Xception probability ≥ 0.9) is RC in Xception model
p-N-ASC_L_S	The proportion of N-ASC_L_S to ASC_L_S with yolov3 probability ≥ 0.0264
p-N-ASC_H_S	The proportion of N-ASC_H_S to ASC_H_S with yolov3 probability ≥ 0.0264
p-N-KC	The proportion of N-KC to KC with yolov3 probability ≥ 0.0264
p-N-ASC_H_S_0.2	The proportion of ASC_H_S with yolov3 probability ≥ 0.2 to ASC_H_S with yolov3 probability ≥ 0.0264
p-N-ASC_L_S_0.2	The proportion of ASC_L_S with yolov3 probability ≥ 0.2 to ASC_L_S with yolov3 probability ≥ 0.0264
p-N-KC_0.2	The proportion of KC with yolov3 probability ≥ 0.2 to KC with yolov3 probability ≥ 0.0264

Supp. Table 05 XGBoost 10-fold cross-validation result in validation-set

Fold	Actual	Negative	Positive	Total	Accuracy
1	Negative	5942	321	6263	0.948746607
	Positive	490	2464	2954	0.834123223
	**Total**	**6432**	**2785**	**9217**	**0.912010416**
2	Negative	5949	314	6263	0.949864282
	Positive	493	2461	2954	0.833107651
	**Total**	**6442**	**2775**	**9217**	**0.912444396**
3	Negative	5916	347	6263	0.944595242
	Positive	453	2501	2954	0.846648612
	**Total**	**6369**	**2848**	**9217**	**0.913203862**
4	Negative	5942	321	6263	0.948746607
	Positive	462	2491	2953	0.843548933
	**Total**	**6404**	**2812**	**9216**	**0.915039063**
5	Negative	5914	349	6263	0.944275906
	Positive	472	2481	2953	0.840162547
	**Total**	**6386**	**2830**	**9216**	**0.910915799**
6	Negative	5915	348	6263	0.944435574
	Positive	466	2487	2953	0.842194379
	**Total**	**6381**	**2835**	**9216**	**0.911675347**
7	Negative	5943	320	6263	0.948906275
	Positive	478	2475	2953	0.838130715
	**Total**	**6421**	**2795**	**9216**	**0.913411458**
8	Negative	5917	345	6262	0.944905781
	Positive	482	2471	2953	0.83677616
	**Total**	**6399**	**2816**	**9215**	**0.910255019**
9	Negative	5916	346	6262	0.944746088
	Positive	461	2492	2953	0.843887572
	**Total**	**6377**	**2838**	**9215**	**0.912425393**
10	Negative	5920	342	6262	0.945384861
	Positive	479	2474	2953	0.837792076
	**Total**	**6399**	**2816**	**9215**	**0.910906131**

Supp. Table 06 Jointly training-set results predicted by 10 models obtained from 10-fold cross-validation

Actual	Negative	Positive	Total	Accuracy
Negative	52268	3814	56082	0.93199244
Positive	0	23566	23566	1
Total	52268	27380	79648	0.952114303

Supp. Table 07 10-fold cross-validation of five classifications XGBoost model for predicting squamous intraepithelial lesions

Fold	Actual	SCC	ASCH	ASCUS	HSIL	LSIL	Total	Accuracy
**1**	SCC	62	4	5	15	3	89	0.696629213
	ASCH	3	200	9	70	7	289	0.692041522
	ASCUS	5	23	981	22	161	1192	0.822986577
	HSIL	6	23	84	562	26	701	0.80171184
	LSIL	3	10	116	24	532	685	0.776642336
	**Total**	**79**	**260**	**1195**	**693**	**729**	**2956**	**0.790595399**
**2**	SCC	55	6	9	15	4	89	0.617977528
	ASCH	1	225	13	46	4	289	0.778546713
	ASCUS	3	21	987	28	153	1192	0.828020134
	HSIL	10	23	85	565	18	701	0.805991441
	LSIL	6	12	115	17	535	685	0.781021898
	**Total**	**75**	**287**	**1209**	**671**	**714**	**2956**	**0.800744249**
**3**	SCC	60	3	6	16	3	88	0.681818182
	ASCH	2	214	14	52	7	289	0.740484429
	ASCUS	3	31	977	37	144	1192	0.819630872
	HSIL	12	30	74	562	23	701	0.80171184
	LSIL	2	13	114	12	544	685	0.794160584
	**Total**	**79**	**291**	**1185**	**679**	**721**	**2955**	**0.797631134**
**4**	SCC	58	5	5	16	4	88	0.659090909
	ASCH	3	221	14	45	6	289	0.764705882
	ASCUS	5	27	982	33	145	1192	0.823825503
	HSIL	11	26	84	564	16	701	0.804564907
	LSIL	5	17	107	24	532	685	0.776642336
	**Total**	**82**	**296**	**1192**	**682**	**703**	**2955**	**0.797631134**
**5**	SCC	61	3	4	16	4	88	0.693181818
	ASCH	3	219	12	49	6	289	0.757785467
	ASCUS	4	25	982	25	156	1192	0.823825503
	HSIL	8	29	64	575	25	701	0.820256776
	LSIL	2	14	104	25	539	684	0.788011696
	**Total**	**78**	**290**	**1166**	**690**	**730**	**2954**	**0.804333108**
**6**	SCC	62	3	2	18	3	88	0.704545455
	ASCH	1	210	19	46	12	288	0.729166667
	ASCUS	5	20	981	34	152	1192	0.822986577
	HSIL	14	18	79	569	21	701	0.811697575
	LSIL	5	17	106	23	533	684	0.779239766
	**Total**	**87**	**268**	**1187**	**690**	**721**	**2953**	**0.797494074**
**7**	SCC	57	6	4	19	2	88	0.647727273
	ASCH	0	226	16	32	14	288	0.784722222
	ASCUS	3	33	979	35	141	1191	0.821998321
	HSIL	9	28	75	564	25	701	0.804564907
	LSIL	2	9	117	25	531	684	0.776315789

	**Total**	**71**	**302**	**1191**	**675**	**713**	**2952**	**0.798441734**
**8**	SCC	56	2	5	22	3	88	0.636363636
	ASCH	3	211	17	46	11	288	0.732638889
	ASCUS	3	28	985	22	153	1191	0.827036104
	HSIL	16	25	83	542	34	700	0.774285714
	LSIL	4	20	101	18	541	684	0.790935673
	**Total**	**82**	**286**	**1191**	**650**	**742**	**2951**	**0.791257201**
**9**	SCC	60	3	7	16	2	88	0.681818182
	ASCH	3	205	20	53	7	288	0.711805556
	ASCUS	0	18	965	41	167	1191	0.810243493
	HSIL	8	29	75	571	17	700	0.815714286
	LSIL	0	16	100	23	545	684	0.796783626
	**Total**	**71**	**271**	**1167**	**704**	**738**	**2951**	**0.794984751**
**10**	SCC	56	8	5	15	4	88	0.636363636
	ASCH	3	213	16	46	10	288	0.739583333
	ASCUS	6	21	969	38	157	1191	0.813602015
	HSIL	4	28	82	566	20	700	0.808571429
	LSIL	3	10	101	25	545	684	0.796783626
	**Total**	**72**	**280**	**1173**	**690**	**736**	**2951**	**0.796001355**

Supp. Table 08 Prediction process of AGC, EMC and infectious lesions

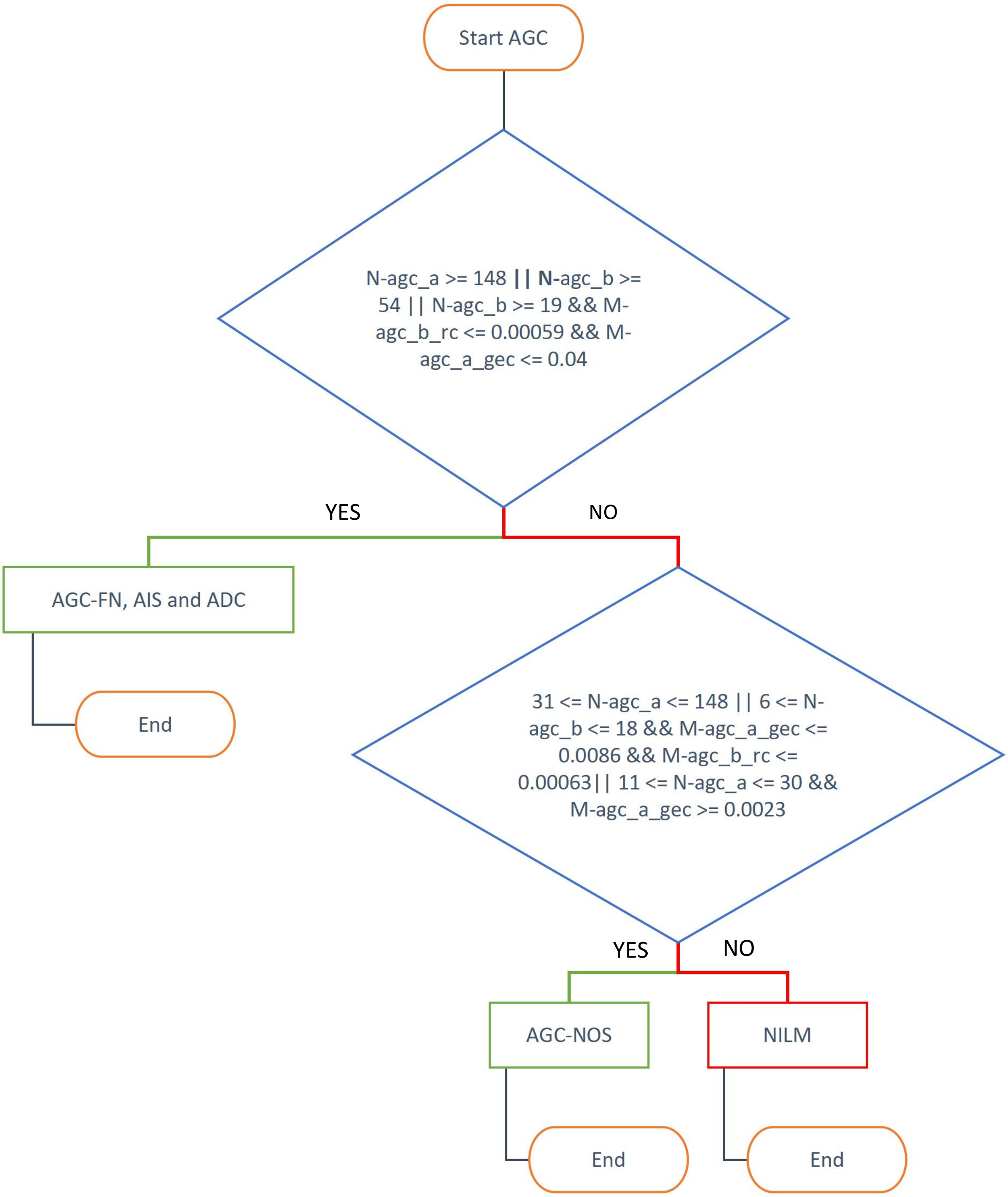

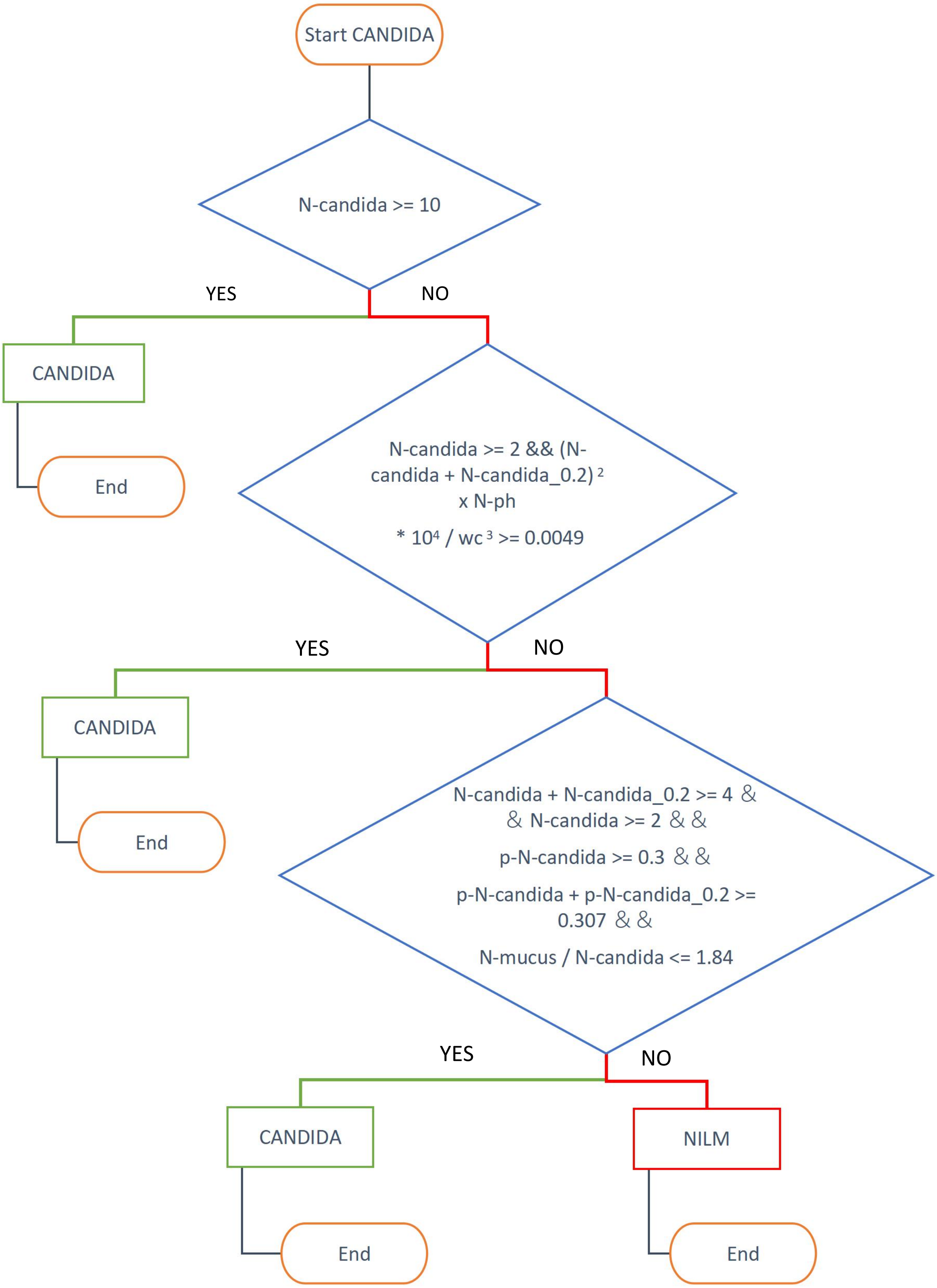
Start CANDIDA
N-candida >= 10
YES
NO
CANDIDA
End
N-candida >= 2 && (N-candida + N-candida_0.2) 2
x N-ph
* 104 / wc 3 >= 0.0049
YES
NO
CANDIDA
End
N-candida + N-candida_0.2 >= 4 &
& N-candida >= 2 & &
p-N-candida >= 0.3 & &
p-N-candida + p-N-candida_0.2 >=
0.307 & &
N-mucus / N-candida <= 1.84
YES
NO
CANDIDA
NILM
End
End

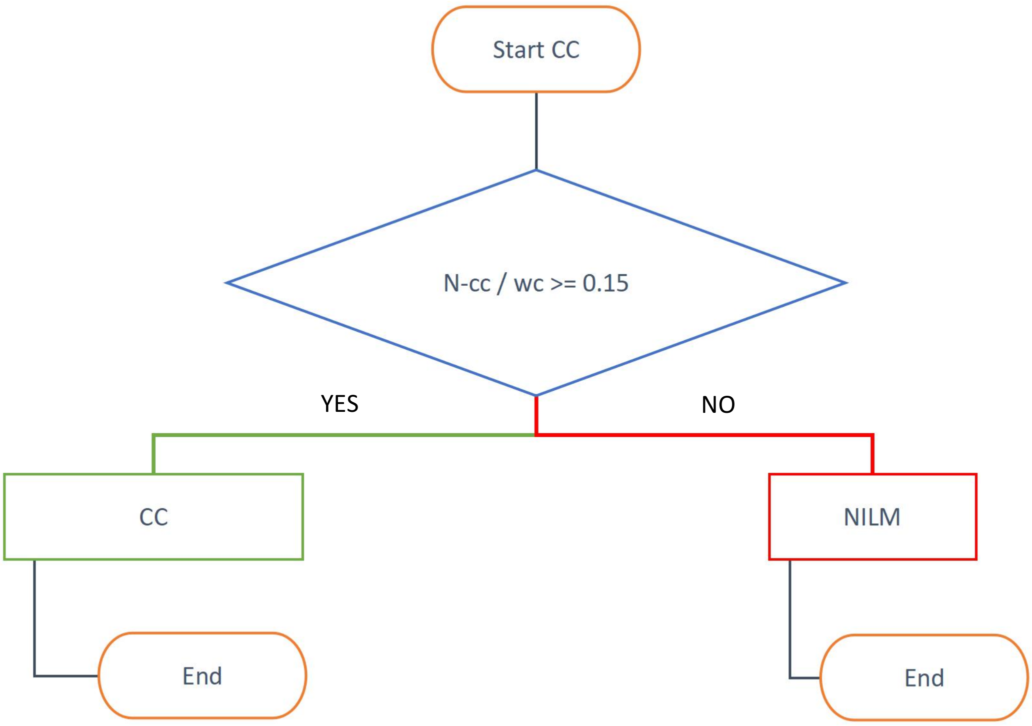
Start CC
N-cc / wc >= 0.15
YES
NO
CC
NILM
End
End

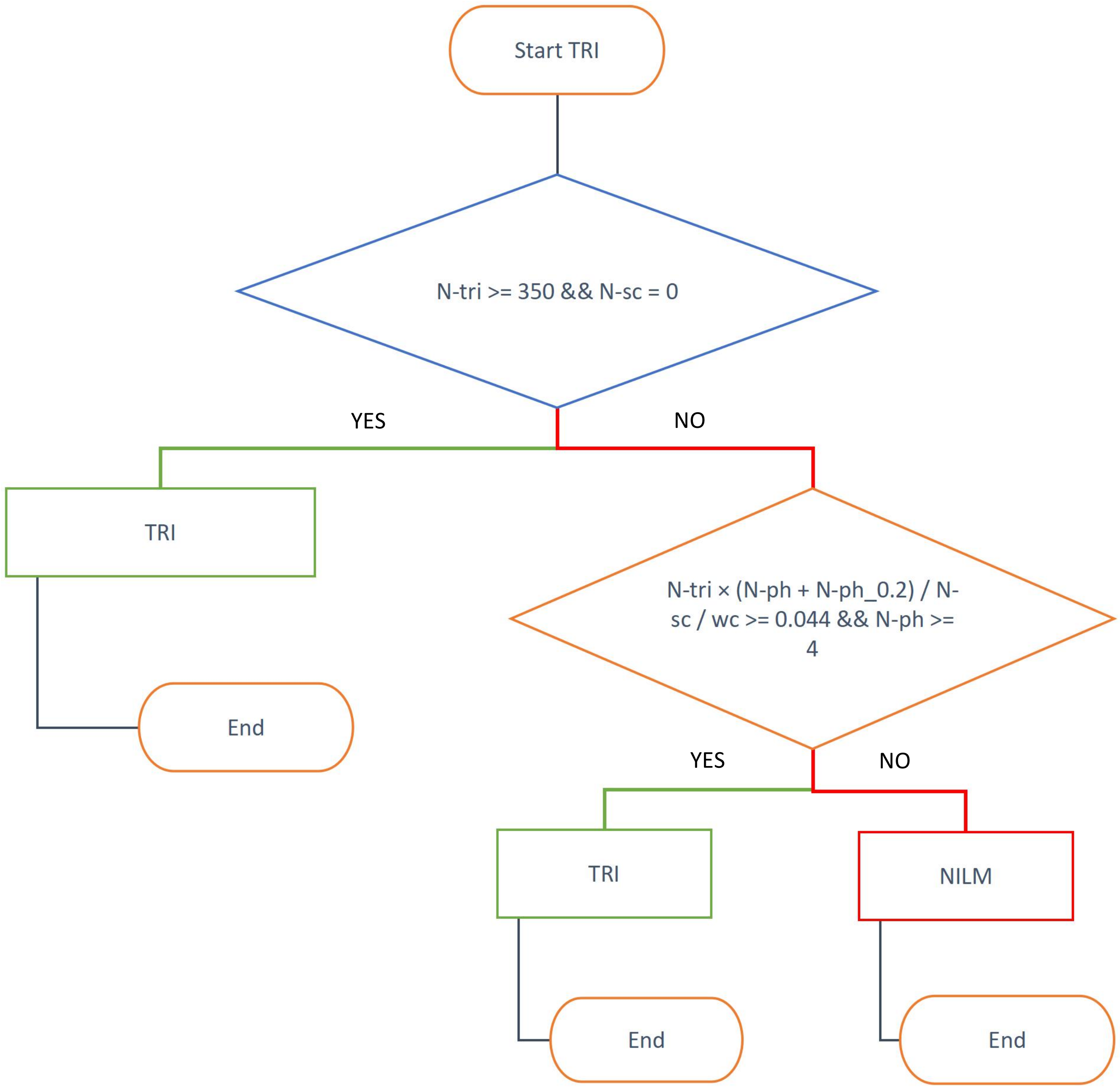
Start TRI
N-tri >= 350 && N-sc = 0
YES
NO
TRI
End
N-tri × (N-ph + N-ph_0.2) / N-sc / wc >= 0.044 && N-ph >= 4
YES
NO
TRI
NILM
End
End

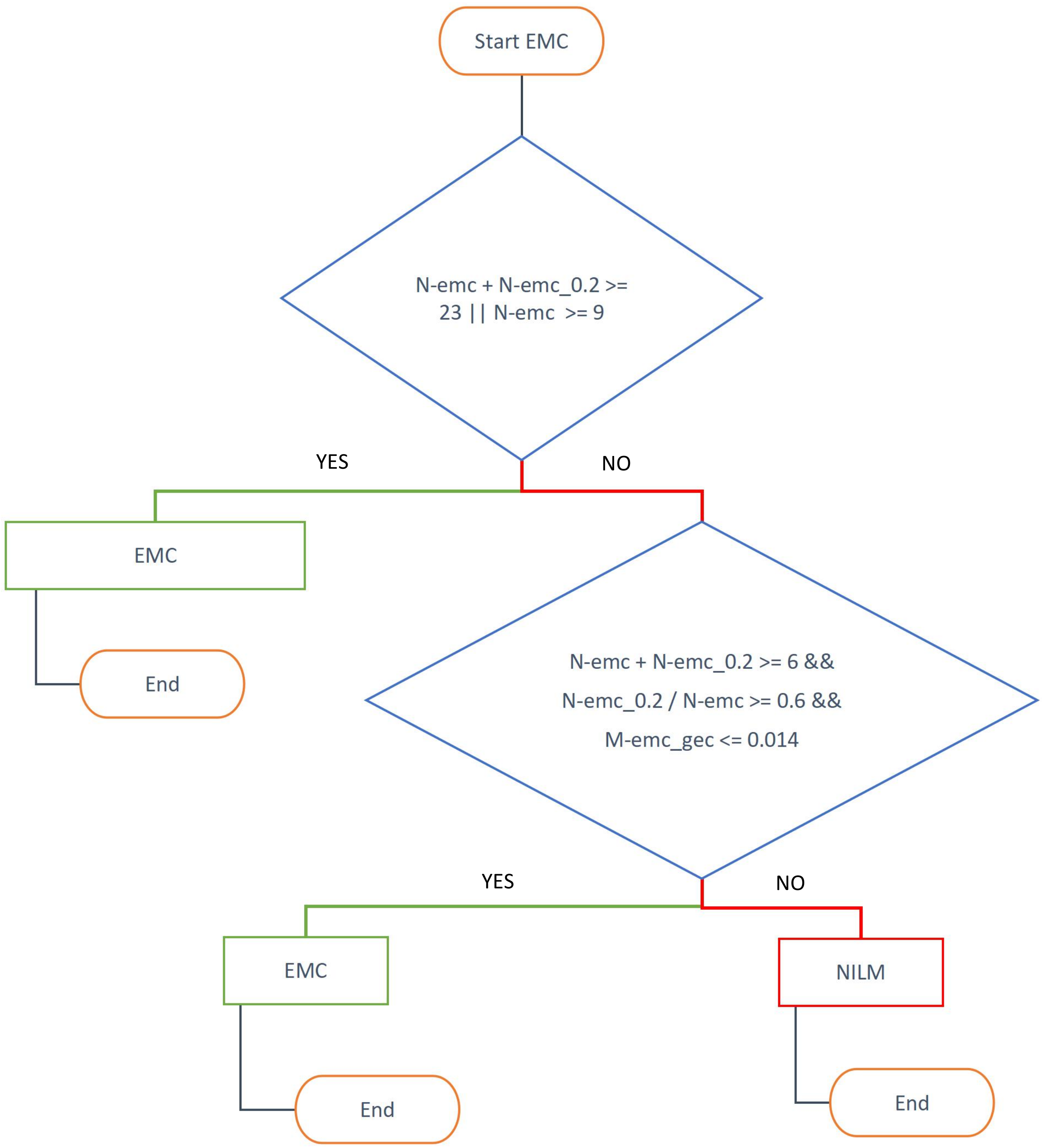
Start EMC
N-emc + N-emc_0.2 >= 23 || N-emc >= 9
YES
NO
EMC
End
N-emc + N-emc_0.2 >= 6 &&
N-emc_0.2 / N-emc >= 0.6 &&
M-emc_gec <= 0.014
YES
NO
EMC
NILM
End
End

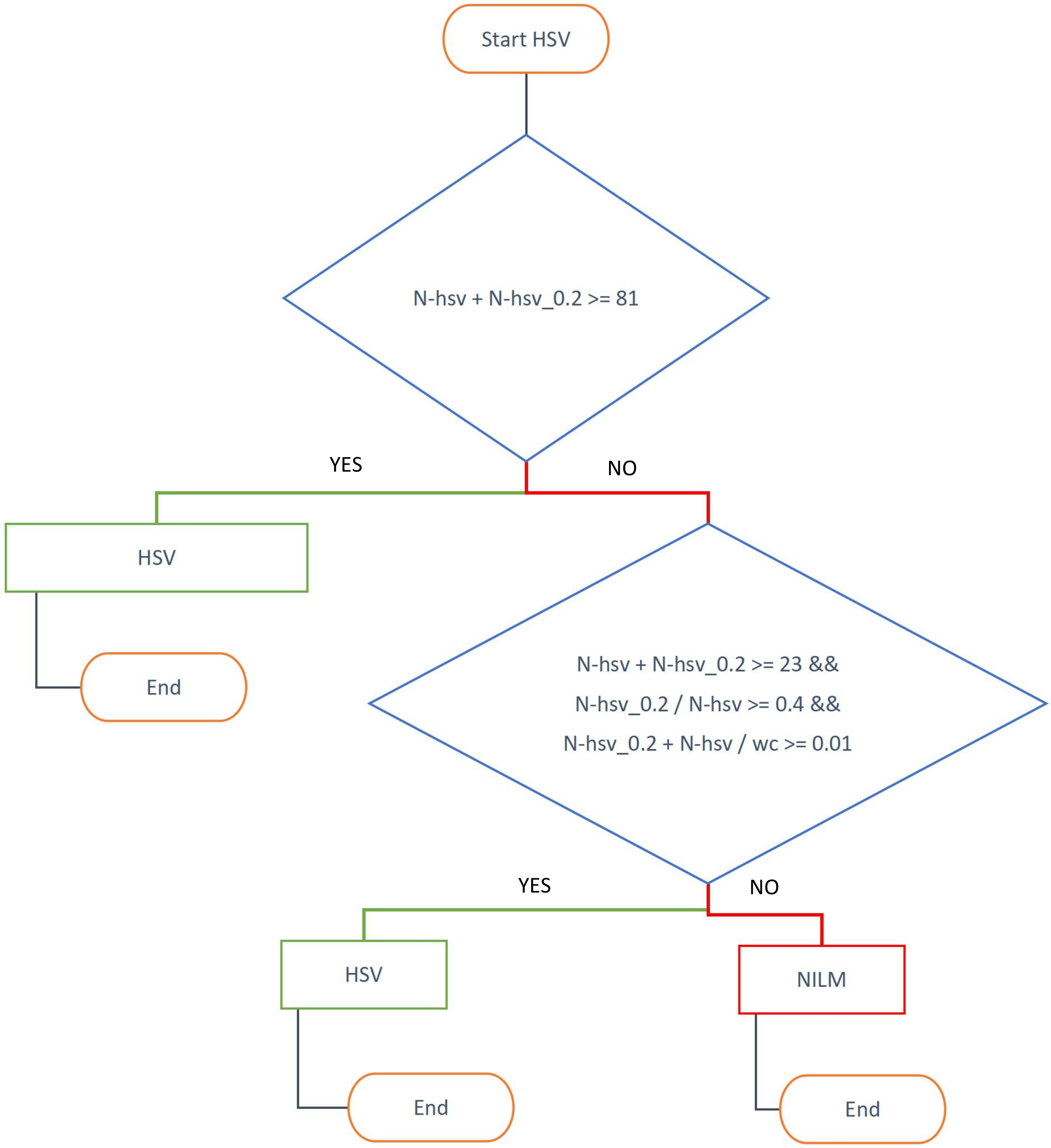

Start HSV
N-hsv + N-hsv_0.2 >= 81
YES
NO
HSV
End
N-hsv + N-hsv_0.2 >= 23 &&
N-hsv_0.2 / N-hsv >= 0.4 &&
N-hsv_0.2 + N-hsv / wc >= 0.01
YES
NO
HSV
NILM
End
End

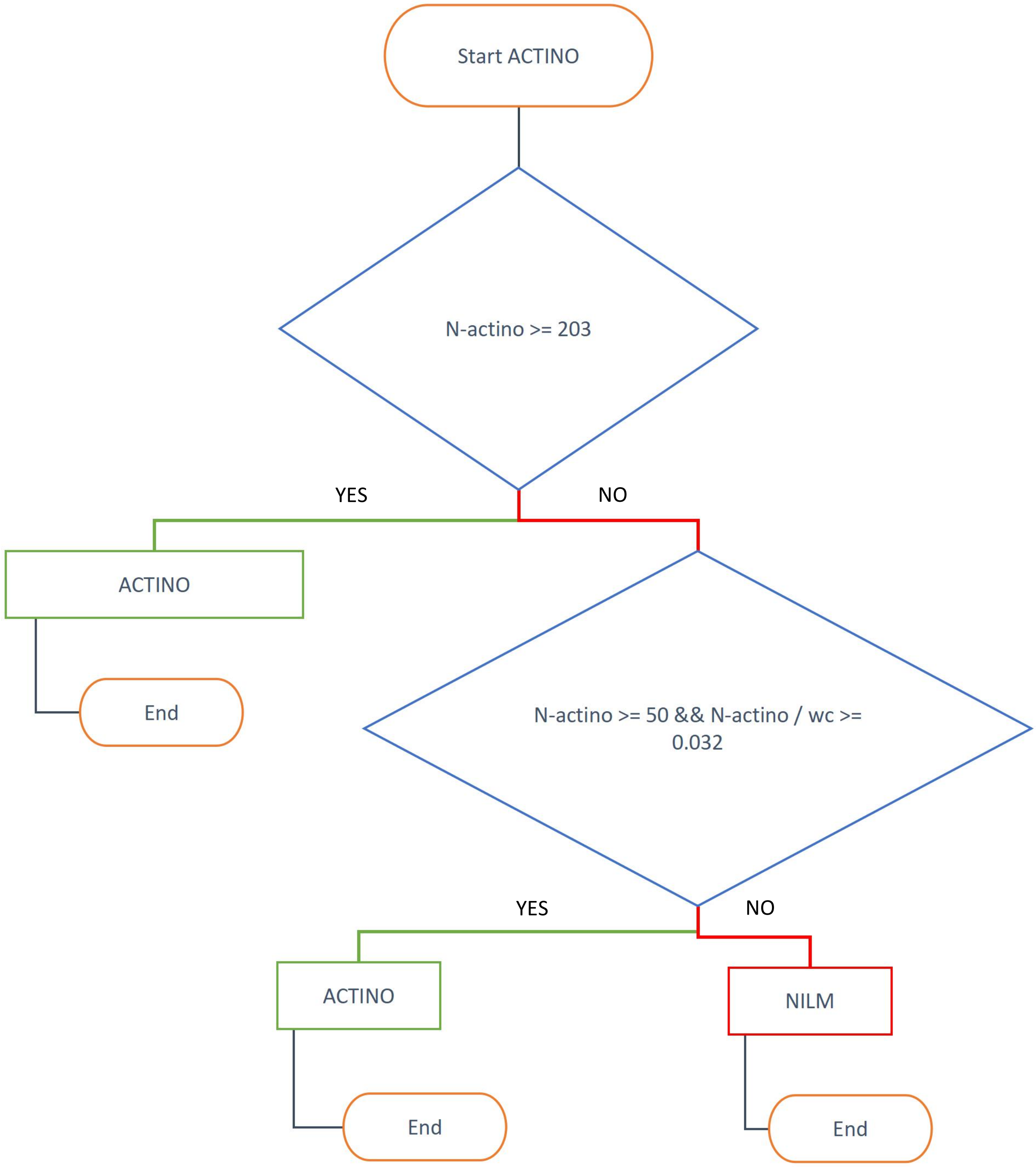

Start ACTINO
N-actino >= 203
YES
NO
ACTINO
End
N-actino >= 50 && N-actino / wc >= 0.032
YES
NO
ACTINO
NILM
End
End

Appendix: annotation of abbreviation

Name	Annotation
N-agc_a	Number of agc_a (with yolov3 probability ≥ 0.0264 and Xception probability ≥ 0.9)
**N-**agc_b	Number of agc_b (with yolov3 probability ≥ 0.0264 and Xception probability ≥ 0.9)
M-agc_b_rc	The median probability that agc_b (with yolov3 probability ≥ 0.0264 and Xception probability ≥ 0.9) is rc in Xception model
M-agc_a_gec	The median probability that agc_a (with yolov3 probability ≥ 0.0264 and Xception probability ≥ 0.9) is gec in Xception model
N-candida	Number of candida (with yolov3 probability ≥ 0.0264 and Xception probability ≥ 0.9)
N-candida_0.2	Number of candida (with yolov3 probability ≥ 0.2)
N-ph	Number of ph (with yolov3 probability ≥ 0.0264 and Xception probability ≥ 0.9)
wc	The number of patches detected by yolov3 model in the WSI
p-N-candida	The proportion of N-candida to candida with yolov3 probability ≥ 0.0264
p-N-candida_0.2	The proportion of candida with yolov3 probability ≥ 0.2 to candida with yolov3 probability ≥ 0.0264
N-mucus	Number of mucus
N-cc	Number of cc (with yolov3 probability ≥ 0.0264 and Xception probability ≥ 0.9)
N-tri	Number of tri (with yolov3 probability ≥ 0.0264 and Xception probability ≥ 0.9)
N-sc	Number of sc (with yolov3 probability ≥ 0.0264 and Xception probability ≥ 0.9)
N-ph_0.2	Number of ph (with yolov3 probability ≥ 0.2)
N-emc	Number of emc (with yolov3 probability ≥ 0.0264 and Xception probability ≥ 0.9)
N-emc_0.2	Number of emc (with yolov3 probability ≥ 0.2)
M-emc_gec	The median probability that emc (with yolov3 probability ≥ 0.0264 and Xception probability ≥ 0.9) is gec in Xception model
N-hsv	Number of hsv (with yolov3 probability ≥ 0.0264 and Xception probability ≥ 0.9)
N-hsv_0.2	Number of hsv (with yolov3 probability ≥ 0.2)
N-actino	Number of actino (with yolov3 probability ≥ 0.0264 and Xception probability ≥ 0.9)

Supp. Table 09 Logical decision trees accuracy of specific TBS classification in retrospective smear samples

TBS classifications	Number	Sensitivity	Specificity
AGC_NOS	271	76.75%	99.13%
AGC_FN, AIS and ADC	285	97.89%	99.44%
TRI	1714	80.98%	97.38%
CAN	6918	85.99%	99.34%
ACTINO	1049	85.99%	99.71%
CC	4531	88.99%	99.92%
HSV	248	87.90%	99.93%
EMC (in women older than 45 years)	405	77.78%	98.65%

Supp. Table 10 XGBoost 10-fold cross-validation of DPIQC system in Training-set

Fold	Actual	Qualified	Unqualified	Total	Accuracy
1	Qualified	43032	177	43209	0.995903631
	Unqualified	275	33011	33286	0.991738268
	**Total**	**43307**	**33188**	**76495**	**0.994091117**
2	Qualified	43037	173	43210	0.995996297
	Unqualified	291	32995	33286	0.991257586
	Total	43328	33168	76496	0.993934323
3	Qualified	43027	183	43210	0.995764869
	Unqualified	293	32993	33286	0.9911975
	**Total**	**43320**	**33176**	**76496**	**0.993777452**
4	Qualified	43035	175	43210	0.995950012
	Unqualified	297	32989	33286	0.99107733
	**Total**	**43332**	**33164**	**76496**	**0.993829743**
5	Qualified	43045	165	43210	0.996181439
	Unqualified	281	33005	33286	0.991558012
	**Total**	**43326**	**33170**	**76496**	**0.99416963**
6	Qualified	43034	176	43210	0.995926869
	Unqualified	294	32993	33287	0.991167723
	**Total**	**43328**	**33169**	**76497**	**0.993855968**
7	Qualified	43022	188	43210	0.995649155
	Unqualified	293	32994	33287	0.991197765
	**Total**	**43315**	**33182**	**76497**	**0.993712172**
8	Qualified	43045	165	43210	0.996181439
	Unqualified	294	32993	33287	0.991167723
	**Total**	**43339**	**33158**	**76497**	**0.993999765**
9	Qualified	43030	180	43210	0.995834298
	Unqualified	277	33010	33287	0.991678433
	**Total**	**43307**	**33190**	**76497**	**0.99402591**
10	Qualified	43031	179	43210	0.99585744
	Unqualified	274	33013	33287	0.991768558
	**Total**	**43305**	**33192**	**76497**	**0.994078199**

Supp. Table 11 XGBoost 10-fold cross-validation of DPIQC system in validation-set

Fold	Actual	Qualified	Unqualified	Total	Accuracy
1	Qualified	4767	35	4802	0.99271137
	Unqualified	42	3657	3699	0.98864558
	**Total**	**4809**	**3692**	**8501**	**0.990942242**
2	Qualified	4772	29	4801	0.993959592
	Unqualified	39	3660	3699	0.98945661
	Total	4811	3689	8500	0.992
3	Qualified	4769	32	4801	0.993334722
	Unqualified	44	3655	3699	0.988104893
	**Total**	**4813**	**3687**	**8500**	**0.991058824**
4	Qualified	4763	38	4801	0.992084982
	Unqualified	46	3653	3699	0.987564207
	**Total**	**4809**	**3691**	**8500**	**0.990117647**
5	Qualified	4766	35	4801	0.992709852
	Unqualified	50	3649	3699	0.986482833
	**Total**	**4816**	**3684**	**8500**	**0.99**
6	Qualified	4779	22	4801	0.995417621
	Unqualified	49	3649	3698	0.986749594
	**Total**	**4828**	**3671**	**8499**	**0.991646076**
7	Qualified	4770	31	4801	0.993543012
	Unqualified	39	3659	3698	0.989453759
	**Total**	**4809**	**3690**	**8499**	**0.991763737**
8	Qualified	4772	29	4801	0.993959592
	Unqualified	46	3652	3698	0.987560844
	Total	4818	3681	8499	0.991175432
9	Qualified	4776	25	4801	0.994792752
	Unqualified	41	3657	3698	0.988912926
	**Total**	**4817**	**3682**	**8499**	**0.992234381**
10	Qualified	4773	28	4801	0.994167882
	Unqualified	54	3644	3698	0.985397512
	**Total**	**4827**	**3672**	**8499**	**0.990351806**

Supp. Table 12 Comparison of digital smears quality control between DPIQC system and Cytologists

**Medical**	**Unqualified number**		**Unqualified rate**		**Unanimous**	**Consistent**
**institutions**	**DPIQC**	**Cytologists**	**DPIQC**	**Cytologists**	**number**	**rate**
A	164	154	0.047344111	0.044457275	138	0.896103896
B	1253	1345	0.14497281	0.155617263	1184	0.880297398
C	312	298	0.052463427	0.050109299	274	0.919463087
D	142	124	0.054636399	0.047710658	112	0.903225806
E	713	651	0.183055199	0.167137356	591	0.907834101
F	90	76	0.053892216	0.045508982	66	0.868421053
G	216	195	0.047619048	0.042989418	174	0.892307692
H	233	246	0.154817276	0.16345515	202	0.821138211
I	219	229	0.09651829	0.100925518	205	0.895196507
J	65	55	0.048945783	0.041415663	51	0.927272727
K	246	236	0.111818182	0.107272727	219	0.927966102
**Total**	**3653**	**3609**	**0.09599012**	**0.094833929**	**3216**	**0.891105569**

Supp. Table 13 Performance of AIATBS system in predicting multi-center prospective smear samples

Type of lesion	TBS classification	Number	Sensitivity	Specificity
**Intraepithelial lesions**	ASCUS	2089	85.59%	90.02%
	LSIL, ASCH, HSIL, SCC	1961	98.83%	98.91%
	AGC_NOS	52	73.01%	98.75%
	AGC_FN, AIS, ADC	60	88.33%	100.00%
	**Total**	**4162**	**92.00%**	**84.39%**
**other lesions**	TRI	721	81.83%	99.16%
	CAN	1204	76.41%	99.34%
	ACTINO	62	85.48%	99.97%
	CC	1479	88.57%	99.33%
	HSV	35	94.29%	99.99%
	EMC	29	82.76%	99.97%
	**Total**	**3530**	**83.00%**	**97.75%**

Supp. Table 14 Performance comparison of AIATBS system in predicting smears obtained from different medical institutions

Medical Institutions	Provided retrospective samples (YES/NO)	Sensitivity		Specificity
		Intraepithelial lesions	Other lesions	
1	YES	0.930875576	0.834983498	0.868345324
2	YES	0.921001927	0.855072464	0.823597551
3	YES	0.901960784	0.841049383	0.800611487
4	YES	0.925170068	0.820433437	0.832913941
11	YES	0.931856899	0.825	0.845714286
**Total (YES)**		**0.924137931**	**0.841888176**	**0.826900367**
8	NO	0.905405405	0.68115942	0.811337467
9	NO	0.933333333	0.781350482	0.831030151
10	NO	0.923076923	0.765625	0.821299639
5	NO	0.896975425	0.880658436	0.737905369
6	NO	0.908163265	0.857142857	0.811874106
7	NO	0.912663755	0.811091854	0.843767786
**Total (NO)**		**0.904705882**	**0.810830861**	**0.813123706**

Supp. Table 15 The confusion matrix of TBS classification of squamous intraepithelial lesions in prospective smear samples

Actual	ASCUS	LSIL	ASCH	HSIL	SCC	Total	Accuracy
**ASCUS**	1443	180	136	27	2	1788	0.80704698
**LSIL**	194	517	17	6	2	736	0.702445652
**ASCH**	102	15	358	95	5	575	0.622608696
**HSIL**	57	18	69	409	21	574	0.712543554
**SCC**	1	0	5	11	36	53	0.679245283
**Total**	1797	730	585	548	66	3726	0.741545894

Supp. Table 16 The influence of different factors on the analysis time of AIATBS system

Methods	Time (s)	p-value
Natural sedimentation	66.27 ± 27.71	< 0.0001
Membrane sedimentation	171.8 ± 38.02	
Centrifugal sedimentation	85.30 ± 31.86	
EA36	67.25 ± 28.67	0.0657
EA50	68.69 ± 34.79	
Scanner 1	89.30 ± 53.15	< 0.0001
Scanner 2	107.2 ± 52.66	

Time is presented in mean ±standard deviation in seconds.

Supp. Table 17 TBS classification accuracy of squamous intraepithelial lesions diagnosed by senior cytologist

Actual	ASCUS	LSIL	ASCH	HSIL	SCC	Total	Accuracy
**ASCUS**	735	95	82	17	0	929	0.791173305
**LSIL**	63	405	12	8	0	488	0.829918033
**ASCH**	19	5	196	56	0	276	0.710144928
**HSIL**	9	7	36	322	2	376	0.856382979
**SCC**	1	0	2	3	27	33	0.818181818
**Total**	827	512	328	406	29	2102	0.801617507

Supp. Table 18 The confusion matrix of TBS classification of squamous intraepithelial lesions predicted by AIATBS system in 6 medical institutions

Actual	ASCUS	LSIL	ASCH	HSIL	SCC	Total	Accuracy
**ASCUS**	824	87	78	19	1	1009	0.816650149
**LSIL**	134	362	12	0	0	508	0.712598425
**ASCH**	49	5	200	29	3	286	0.699300699
**HSIL**	35	13	64	270	10	392	0.68877551
**SCC**	1	0	3	9	21	34	0.617647059
**Total**	1043	467	357	327	35	2229	0.752355316

Supp. Table 19 Server hardware configuration of AIATBS system

Accessories	Specifications	Number
CPU	I7-7700K 2.9GHz 8 core 16 thread	1
GPU	RTX 2080	2
RAM	Kingston DDR4 16G	2
SSD	SAMSUNG 970EVO 500GB M.2	1
Mainboard	ASUS Z490	1
Power Supply	USCORSAIR HX1000	1

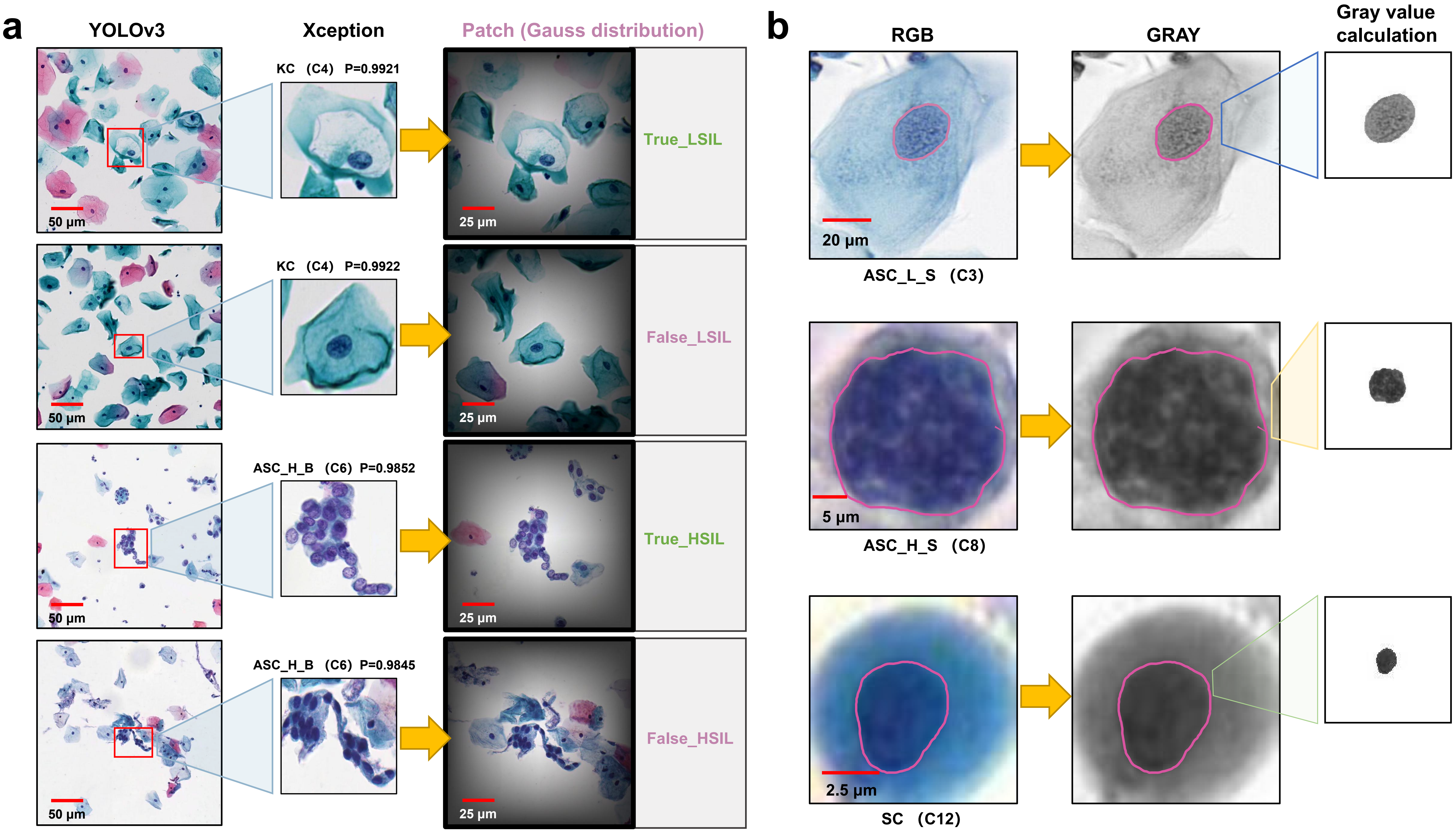
a
YOLOv3
Xception
Patch (Gauss distribution)
KC (C4) P=0.9921
True_LSIL
KC (C4) P=0.9922
False_LSIL
ASC_H_B (C6) P=0.9852
True_HSIL
ASC_H_B (C6) P=0.9845
False_HSIL
50 μm
25 μm
b
RGB
GRAY
Gray value calculation
20 μm
ASC_L_S (C3)
5 μm
ASC_H_S (C8)
2.5 μm
SC (C12)

Supp. Figure 1. Further detailed identification for false targets of squamous intraepithelial lesions. (a). Application process of patch model: First, the YOLOv3 model detected targets; second, the Xception model classified the targets but may have misjudged the false positive targets with high probability; finally, we extracted the classification probability of false positive targets from the Patch model (introduce Gaussian distribution) for further identification. In this Figure, P refers to the target classification probability predicted by Xception model. **(b).** The process of cell nucleus segmentation and calculation of nuclear gray value.

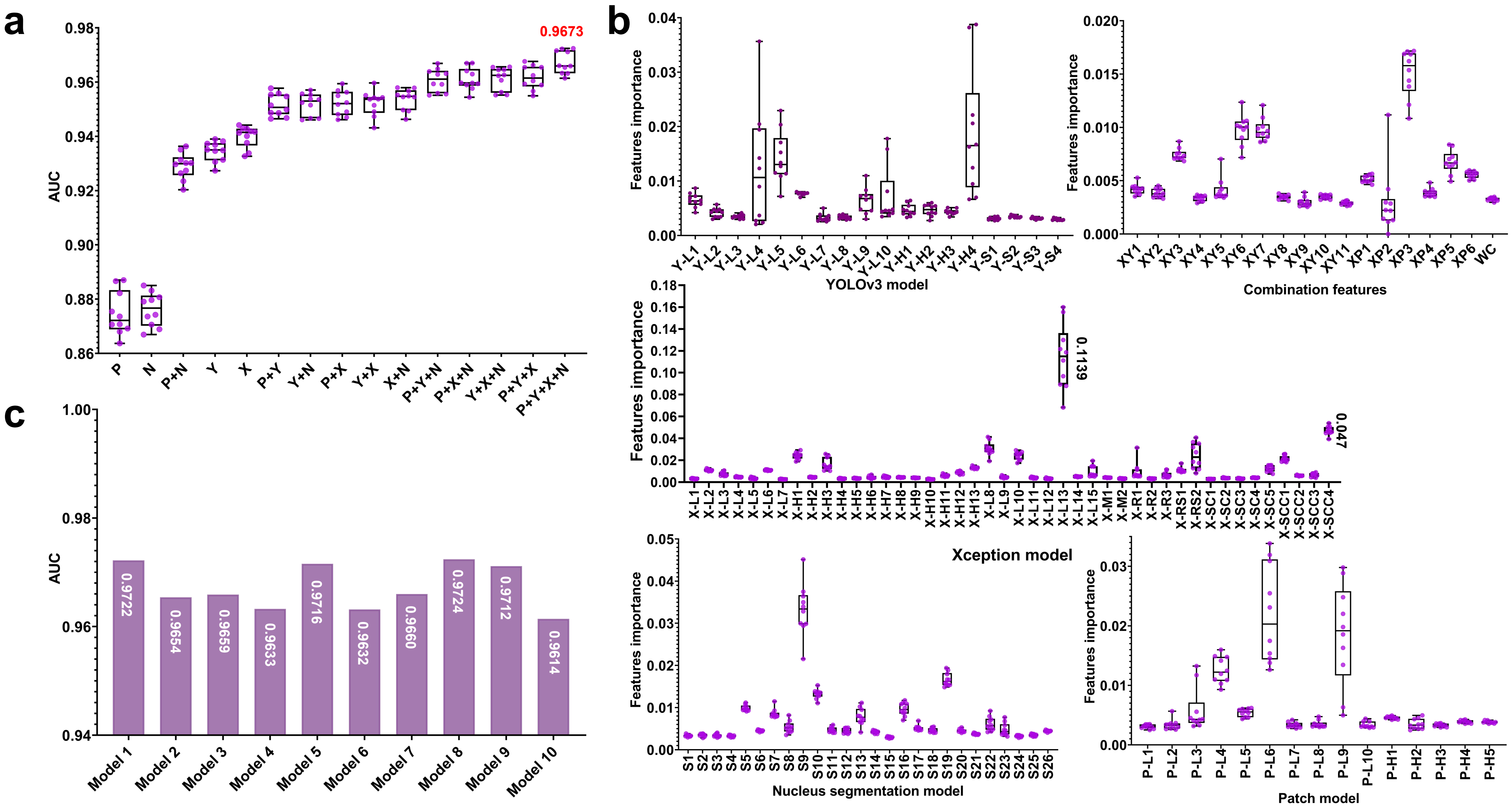

a
0.9673
AUC
P
N
P+N
Y
X
P+Y
Y+N
P+X
Y+X
X+N
P+Y+N
P+X+N
Y+X+N
P+Y+X
P+Y+X+N
b
Features importance
YOLOv3 model
Combination features
0.1139
0.047
Xception model
Nucleus segmentation model
Patch model
c
AUC
0.9722
0.9654
0.9659
0.9633
0.9716
0.9632
0.9660
0.9724
0.9712
0.9614
Model 1
Model 2
Model 3
Model 4
Model 5
Model 6
Model 7
Model 8
Model 9
Model 10

Supp. Figure 2. XGBoost model can efficiently fit the features extracted from multiple deep learning models. (a) AUC values (Mean ± standard deviation, n = 10) of squamous intraepithelial lesions predicted by XGBoost model in different model combinations (Y: YOLOv3 model, X: Xception model, P: Patch model, N: Nucleus segmentation model, +: combination). **(b)**. The importance of features (Mean ± standard deviation, n = 10) provided by different deep learning models in XGBoost model (Y-: YOLOv3 model features, X-: Xception model features, P-: Patch model features, S-: Nucleus segmentation model features). **(c).** AUC values of 10 models obtained from 10-fold cross-validation in the optimal XGBoost model.

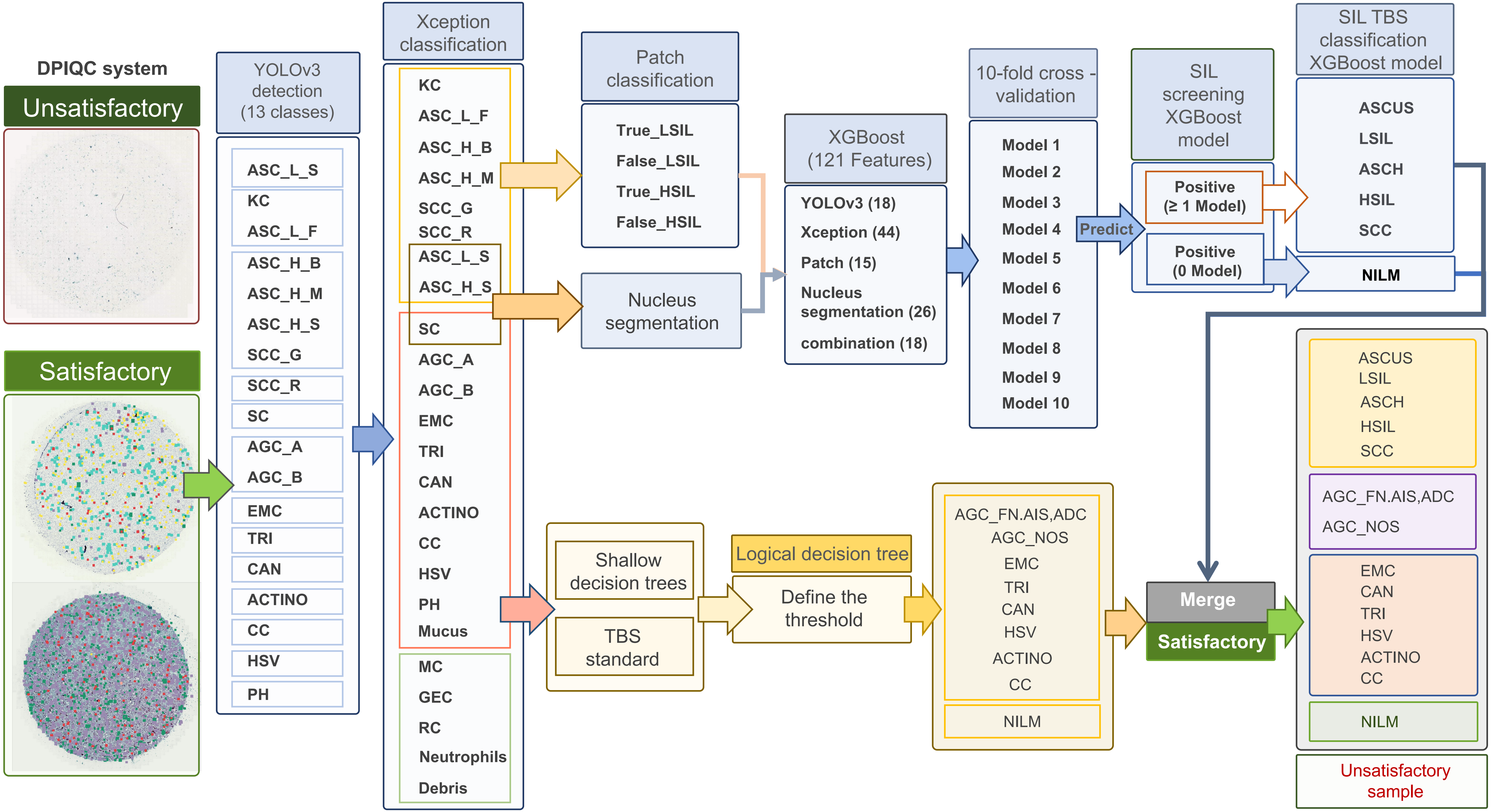

DPIQC system
Unsatisfactory
Satisfactory
YOLOv3 detection (13 classes)
ASC_L_S
KC
ASC_L_F
ASC_H_B
ASC_H_M
ASC_H_S
SCC_G
SCC_R
SC
AGC_A
AGC_B
EMC
TRI
CAN
ACTINO
CC
HSV
PH
Xception classification
KC
ASC_L_F
ASC_H_B
ASC_H_M
SCC_G
SCC_R
ASC_L_S
ASC_H_S
SC
AGC_A
AGC_B
EMC
TRI
CAN
ACTINO
CC
HSV
PH
Mucus
MC
GEC
RC
Neutrophils
Debris
Patch classification
True_LSIL
False_LSIL
True_HSIL
False_HSIL
Nucleus segmentation
XGBoost (121 Features)
YOLOv3 (18)
Xception (44)
Patch (15)
Nucleus segmentation (26)
combination (18)
10-fold cross - validation
Model 1
Model 2
Model 3
Model 4
Model 5
Model 6
Model 7
Model 8
Model 9
Model 10
Predict
SIL screening XGBoost model
Positive (≥ 1 Model)
Positive (0 Model)
SIL TBS classification XGBoost model
ASCUS
LSIL
ASCH
HSIL
SCC
NILM
Shallow decision trees
TBS standard
Logical decision tree
Define the threshold
AGC_FN.AIS,ADC
AGC_NOS
EMC
TRI
CAN
HSV
ACTINO
CC
NILM
Merge
Satisfactory
ASCUS
LSIL
ASCH
HSIL
SCC
AGC_FN.AIS,ADC
AGC_NOS
EMC
CAN
TRI
HSV
ACTINO
CC
NILM
Unsatisfactory sample

Supp. Figure 3. Flowchart of the AIATBS system. The digital pathology image quality control (DPIQC) system controlled the quality of digital smears. → YOLOv3 model detected all 13 classes from the satisfactory smear. → Xception model classified the targets detected from the YOLOv3 model to 24 classes. → Diagnostic decision pipeline: For squamous intraepithelial lesions (SIL), 8 and 3 relevant targets in Xception model output were fed to the Patch classification model and the Nucleus segmentation model, respectively, for prediction; a total of 121 features were obtained using the classification and probability information output by the four models mentioned above; the SIL screening XGBoost model was trained using those 121 features and validated using 10-fold cross validation; the positivity of a smear was determined by the combination of 10 models of cross validation; negative smear was NILM while the positive ones would be further classified as one of five SIL TBS categories by SIL TBS classification XGBoost model. For other TBS categories (non-SIL), the classification and probability of relevant targets were extracted from the YOLOv3 and Xception model; a logical decision tree classifier that integrated shallow decision trees and TBS standards was used to determine the diagnostic threshold of non-SIL TBS categories; negative smears were NILM while the positive ones would be classified as one of eight non-SIL TBS categories.) → The digital smear final diagnosis result was the combination of the results of the above two diagnostic decision pipelines and DPIQC system.

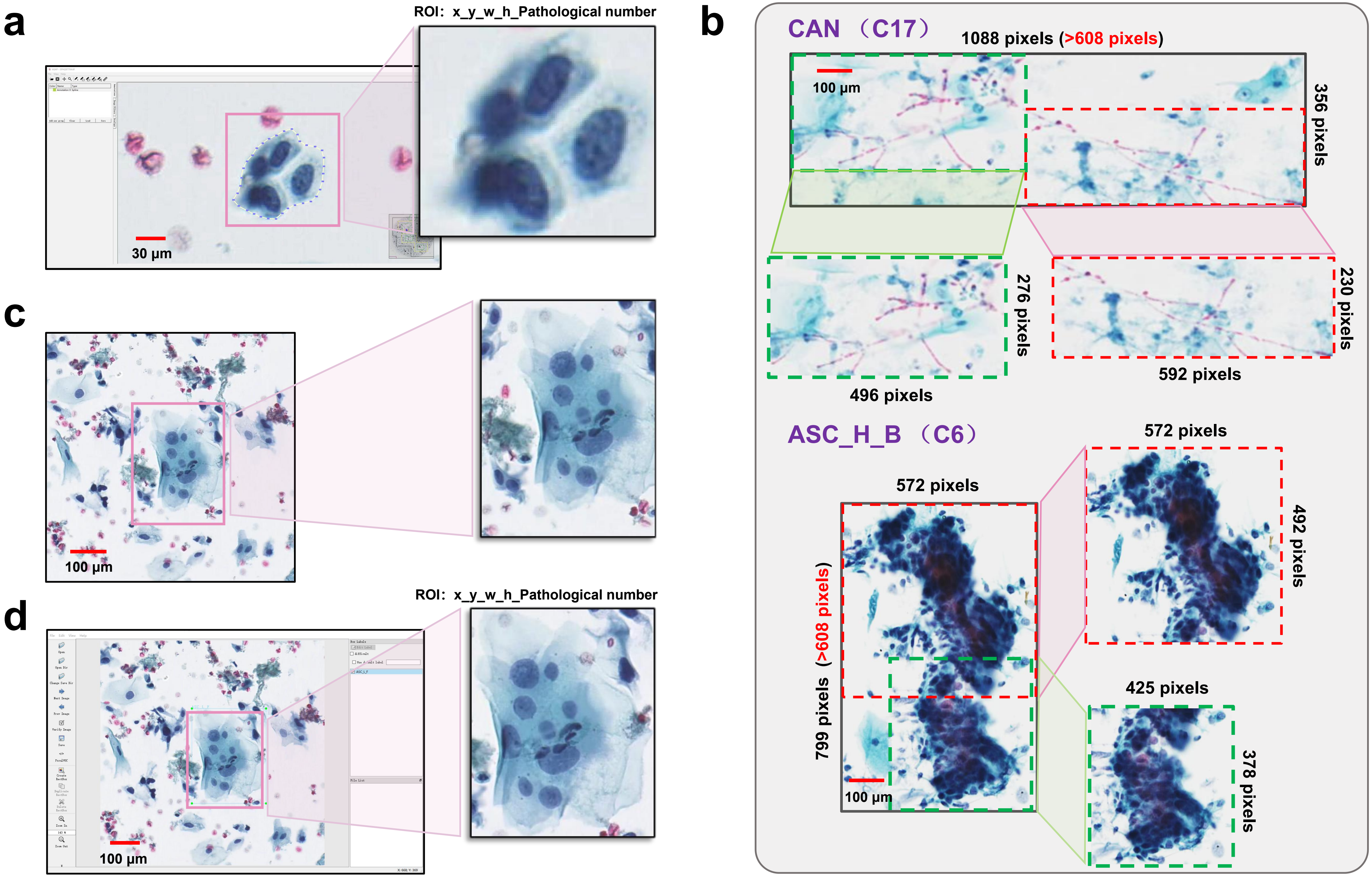
a
ROI: x_y_w_h_Pathological number
30 µm
b
CAN (C17)
1088 pixels (>608 pixels)
100 µm
356 pixels
276 pixels
230 pixels
496 pixels
592 pixels
ASC_H_B (C6)
572 pixels
572 pixels
492 pixels
799 pixels (>608 pixels)
425 pixels
378 pixels
100 µm
c
100 µm
d
ROI: x_y_w_h_Pathological number
100 µm

Supp. Figure 4. Annotation of training data for deep learning models. (a). ASAP 1.8 was used to annotate the boundary of the lesions, and the corresponding ROIs (included the coordinates, width, height and pathological number) were obtained as well. **(b).** Segmented and labeled lesions where the longer edge of images exceeds 608 pixels. **(c).** A 608*608 pixel image, whose center was a smaller image reviewed by the cytologist, was cropped from the WSI, but the ROI of the image was not correct. **(d).** The ROI of the lesion was calibrated using labeling software; The representative images in **(a)-(c)** are from the annotation process.
