# Supplementary material for: Hybrid AI-assistive diagnostic model permits rapid TBS classification of cervical liquid-based thin-layer cell smears
Source: Nat Commun. 2021 Jun 10;12:3541. doi: 10.1038/s41467-021-23913-3 (PMC8192526; doi:10.1038/s41467-021-23913-3)
Supplement: Supplementary file 2 — Reporting Summary [file 41467_2021_23913_MOESM2_ESM.pdf]

nature research

Corresponding author(s): Yanqing Ding
Last updated by author(s): Mar 26, 2021

Reporting Summary

Nature Research wishes to improve the reproducibility of the work that we publish. This form provides structure for consistency and transparency in reporting. For further information on Nature Research policies, see our Editorial Policies and the Editorial Policy Checklist.

Statistics

For all statistical analyses, confirm that the following items are present in the figure legend, table legend, main text, or Methods section.

n/a	Confirmed	
☐	☒	The exact sample size (*n*) for each experimental group/condition, given as a discrete number and unit of measurement
☐	☒	A statement on whether measurements were taken from distinct samples or whether the same sample was measured repeatedly
☐	☒	The statistical test(s) used AND whether they are one- or two-sided<br>*Only common tests should be described solely by name; describe more complex techniques in the Methods section.*
☒	☐	A description of all covariates tested
☒	☐	A description of any assumptions or corrections, such as tests of normality and adjustment for multiple comparisons
☐	☒	A full description of the statistical parameters including central tendency (e.g. means) or other basic estimates (e.g. regression coefficient) AND variation (e.g. standard deviation) or associated estimates of uncertainty (e.g. confidence intervals)
☒	☐	For null hypothesis testing, the test statistic (e.g. *F*, *t*, *r*) with confidence intervals, effect sizes, degrees of freedom and *P* value noted<br>*Give P values as exact values whenever suitable.*
☒	☐	For Bayesian analysis, information on the choice of priors and Markov chain Monte Carlo settings
☒	☐	For hierarchical and complex designs, identification of the appropriate level for tests and full reporting of outcomes
☒	☐	Estimates of effect sizes (e.g. Cohen's *d*, Pearson's *r*), indicating how they were calculated

Our web collection on statistics for biologists contains articles on many of the points above.

Software and code

Policy information about availability of computer code

Data collection	Cervical liquid-based thin-layer cell smears were digitized with two different scanners at 0.25um/pixel resolution.
Data analysis	The code was written in Python 3.6. We used Open Slide(0.4.0), a C library that provided a simple interface, to read whole-slide images. We built YOLOv3, Xception, DenseNet-50, U-Net and XGBoost models to server different purposes, such as target detection, target classification, patch classification, nucleus segmentation, and TBS diagnosis, respectively. We used TensorFlow (1.13.1), Mxnet (1.5.1) to train deep learning models and do network inference. The training code base for the deep learning framework is available at: https://gigantum.com/louguei/ncomms.This framework is general and can be applied to other organs. The core components of the inference system are available at: https://gigantum.com/louguei/ncomms.

For manuscripts utilizing custom algorithms or software that are central to the research but not yet described in published literature, software must be made available to editors and reviewers. We strongly encourage code deposition in a community repository (e.g. GitHub). See the Nature Research guidelines for submitting code & software for further information.

Data

Policy information about availability of data

All manuscripts must include a data availability statement. This statement should provide the following information, where applicable:

- Accession codes, unique identifiers, or web links for publicly available datasets
- A list of figures that have associated raw data
- A description of any restrictions on data availability

The datasets generated during and/or analysed during the current study are not publicly available due to sample information protection, patient privacy protection and medical institutional data regulatory policy but are available from the corresponding author on reasonable request.

Field-specific reporting

Please select the one below that is the best fit for your research. If you are not sure, read the appropriate sections before making your selection.

- [x] Life sciences
- [ ] Behavioural & social sciences
- [ ] Ecological, evolutionary & environmental sciences

For a reference copy of the document with all sections, see nature.com/documents/nr-reporting-summary-flat.pdf

Life sciences study design

All studies must disclose on these points even when the disclosure is negative.

Sample size	We collected as many samples as possible under the conditions of the existing time, personnel and the number of scanners. A total of 81,727 cervical liquid-based thin-layer cell smear samples, were collected from five large medical institutes and other anonymized smaller medical institutions within 3 years before May 31, 2019 as our Multi-center retrospective training sample set. The prospective clinical samples (n=34403) between August 1, 2019 and April 30, 2020 from 11 anonymized medical institutions were collected for our clinical validation. Our dataset contains a large amount of samples from multiple institutions covering various subtypes and lesion morphologies, it is hence sufficient for this study.
Data exclusions	Exclude samples with unsatisfactory production staining and scanning.
Replication	For models training, the data of all models were divided into training set, validation set and testing set. Confusion matrix and cross validation (10 or 5 fold) were used to evaluate the performance of the models. Evaluation indicators included Mean Average Precision (mAP), accuracy and Mean Intersection Over Union (mIOU), etc. In addition, we repeated the performance of AIATBS system in the multi-center prospective dataset, which contains samples from 11 medical institutions, three production methods, two kinds of Pap staining and two types of digital scanners. All the results can be repeated.
Randomization	In models training, datasets of different categories were randomly assigned to training set, validation set and test set.
Blinding	In the retrospective experiments, we tried to collect as many samples and datasets as possible. The training of the models followed the principle of randomness. So, blinding was not necessary. In the prospective experiments, the cytopathologist and the AIATBS system were double-blinded and did not interact with each other.

Reporting for specific materials, systems and methods

We require information from authors about some types of materials, experimental systems and methods used in many studies. Here, indicate whether each material, system or method listed is relevant to your study. If you are not sure if a list item applies to your research, read the appropriate section before selecting a response.

Materials & experimental systems

n/a	Involved in the study
[x]	[ ] Antibodies
[x]	[ ] Eukaryotic cell lines
[x]	[ ] Palaeontology and archaeology
[x]	[ ] Animals and other organisms
[x]	[ ] Human research participants
[x]	[ ] Clinical data
[x]	[ ] Dual use research of concern

Methods

n/a	Involved in the study
[x]	[ ] ChIP-seq
[x]	[ ] Flow cytometry
[x]	[ ] MRI-based neuroimaging
